# Small Cell Ovarian Cancer in Adolescents: Report of Two Cases and Review of the Literature

**DOI:** 10.1155/2011/749516

**Published:** 2011-05-31

**Authors:** M. Rovithi, A. G. Pallis, A. Kalykaki, E. Lagoudaki, L. Giannikaki, E. N. Stathopoulos, K. Relakis, V. Georgoulias

**Affiliations:** ^1^Department of Medical Oncology, University General Hospital of Heraklion, P.O. BOX 1352, 711 10 Heraklion, Crete, Greece; ^2^Department of Pathology-Cytopathology, School of Medicine, University of Crete, 711 10 Heraklion, Crete, Greece; ^3^Department of Obstetrics and Gynecology, University General Hospital of Heraklion, 711 10 Heraklion, Crete, Greece

## Abstract

Ovarian small cell carcinoma is a rare and highly malignant neoplasm carrying a poor prognosis. Although combination chemotherapy remains the cornerstone of treatment due to the rarity of these tumors, no regimen can be recommended as standard of care although in the majority of cases platinum-based regimens are used. Herein, we report two cases of small cell carcinoma of the ovaries along with a review of the relevant literature.

## 1. Introduction

Ovarian small cell carcinoma is a rare and highly malignant neoplasm that typically occurs in young females. It is generally unilateral. Patients often present with abdominal discomfort, and two thirds of the cases are associated with hypercalcemia. Because of the rareness of this disease, there are no evidence-based treatment recommendations (Table 1). Herein, we report two cases of ovarian small cell carcinoma. We also discuss the existing bibliography regarding its clinical presentation and treatment.

## 2. Case Reports

**Case 1**

The first patient, aged 16, presented with a 2-week abdominal pain. Computed tomographic (CT) scan of the abdomen and pelvis revealed the presence of a complex adnexal mass (10 × 11 × 14.5 cm), seemingly arising from the left ovary while peritoneal fluid was also present. The laboratory tests performed were within normal range except elevated serum levels of LDH. No hypercalcemia was noted. Preoperatively, the serum Ca125 level was 172 U/mL. There was no radiographic evidence of distant metastases. A biopsy was performed, indicative of a stromal neoplasm and particularly a granulosa cell tumor. Due to the rapidly growing tumor, the patient developed obstructive anuria, necessitating the placement of a pig-tail-catheter.

She underwent exploratory laparotomy with suboptimal tumor excision including total abdominal hysterectomy, bilateral salpingo-oophorectomy, omentectomy, excision of part of the ileum, and the infiltrated sigmoid colon. The tumor was located on the surface of the right ovary, infiltrated the fat of the small intestine, the muscle tissue of the colon, the exterior surface of the uterus, and the connective to left ovary tissue. Perceptive residual disease remained in Douglas area and in enlarged para-aortic lymph nodes. The resected left ovarian mass measured grossly 17.5 cm and had a tan-white, lobulated external surface, and solid and cystic areas with focal necrosis. Microscopically, the tumor showed closely packed, small but mostly large cells, with abundant cytoplasm, prominent nucleoli, and little cell differentiation. Extended areas of necrosis were present and mitotic figures were frequent (more than 20 mitoses/10 HPF). Immunohistochemical stains showed strong staining of tumor cells with antibodies to WT1, weak focal staining to Pan CK, CK7, EMA, vimentin, calretin, and CD99. Those characteristics set the diagnosis of small cell ovarian cancer, the large cell variant.

The patient was diagnosed with stage IIIC disease and was referred for chemotherapy. She received 3 cycles of chemotherapy with BEP (bleomycin, etoposide, cisplatin), with relatively good tolerance, beside hematologic toxicity (afebrile gr IV neutropenia, gr I anemia). Due to local disease progression, the regimen was altered to irinotecan and liposomal doxorubicin. She received one cycle with no response. She developed bowel obstruction and despite palliative radiotherapy (2000 cGy with abdominal bath, 150 cGy per day, five days a week), to which she showed some early clinical response, clear progressive disease could be documented. She died six months after the initial diagnosis.

**Case 2**

The second patient, aged 19, presented with a 4-week abdominal pain and constipation, before her admittance with bowel obstruction. A palpable mass was prominent in the lower abdomen and radiographic tests (chest, abdomen and pelvis CT scan) revealed a mass in the lower pelvis obstructing the ureters bilaterally, causing distal distention, probably infiltrated the rectum and dislocated the uterus. Colonoscopy showed mucosal ulcers and pressure from the outside on the rectosigmoid area. Laboratory tests, including serum calcium measurement, were also within normal levels except elevated serum levels of LDH. Preoperatively, Ca125 was 71.4 U/mL. She underwent exploratory laparotomy that revealed free peritoneal fluid and a right ovarian mass infiltrating the colon. Suboptimal mass excision, total abdominal hysterectomy, bilateral salpingo-oophorectomy, omentectomy, and appendectomy were performed; in addition, low anterior resection and colostomy were performed. Postsurgically, perceptible evidence of disease remained locally and in the left parametrium area.

Gross examination showed a large (14 × 13 × 10.5 cm), solid, pale brown mass, infiltrating the omentum, the outer surface of the uterus, and part of the sigmoid. Microscopic examination showed medium sized cells, with round nuclei, prominent nucleoli, and eosinophilic cytoplasm while some contained intracytoplasmic eosinophilic globules. Tumor cells mostly grew in a diffuse pattern. There was brisk mitotic activity (>17 mitoses/10 HPF) and foci of necrosis. Occasional follicle-like spaces containing eosinophilic material were present. Vascular invasion and surface involvement was noted. Immunochemical studies revealed the tumor cells were immunoreactive for vimentin and focally immunoreactive for EMA, and pan-cytokeratin. Furthermore, immunoreactivity for p53 was documented in more than 70% of the tumor cells. The diagnosis was small cell tumor of the ovary.

The patient was referred for chemotherapy and, initially, received 2 cycles of BEP, with moderate tolerance (episode of gr IV neutropenia that demanded the prophylactic use of GCSF, gr II anemia that responded well to rh-EPO, gr IV thrombocytopenia). The patient achieved stable disease as her best response, on the basis of CT scans, but due to clinical benefit and decrease of the Ca125 serum levels, 3 more cycles of etoposide/cisplatin doublet were administered (bleomycin was removed from the regimen due to poor tolerance). Three weeks after the end of chemotherapy, the patient developed bowel obstruction and symptomatic hypercalcemia which was treated with zolendronic acid. CT scans revealed disease progression in the liver with new lesions and local recurrence in the lower abdomen. Second-line chemotherapy with irinotecan and liposomal doxorubicin was administered twice, before the patient developed rectal hemorrhage because of local infiltration and palliative radiotherapy was initiated. The patient died 7 months after the initial diagnosis.

## 3. Background

Small cell carcinoma of the ovary (hypercalcemic type, SCC-HT) is a rare and highly malignant tumor. It generally occurs in young women between 10 and 40 years of age (mean age, 23 years) [1]. Less than 30% of tumors develop in patients younger than 20 years and <1% develop in children. The youngest patient reported to date has been 14 months [1, 10]. It is usually unilateral (99%), but bilateral familial cases have been reported [11]. Since its first description in 1982 by Dickersin and colleagues [12], approximately 190 patients have been reported with OSCCHT—the largest series reported to date included 150 patients [1]. Approximately, 66% of cases are associated with paraneoplastic hypercalcaemia in the genesis of which the secretion by the tumour cells of parathyroid hormone-related protein has been suggested [1, 2, 13]. This phenomenon also provides a convenient biochemical marker, useful for followup.

The differential diagnosis is broad and includes adult and juvenile granulosa cell tumor, primitive germ cell tumor, malignant lymphoma, primitive neuroectodermal tumor, neuroblastoma, desmoplastic small round cell tumor, small cell carcinoma (pulmonary type), and metastatic small cell carcinoma.

The most common microscopic pattern is a sheet-like arrangement of small, closely packed epithelial cells. A helpful, but often very focal, finding in 80% of cases is variably sized follicles that contain eosinophilic fluid or are empty. The most common difference from this pattern, present in about half the tumors, is a component of large cells that usually represents a minor to moderate component of the tumor, but rarely can be the predominant or even exclusive component, leading to use of the descriptor “large cell variant” [1], the variant observed in our first patient.

The histogenesis of small cell carcinoma of ovary is unknown. The tumor cells cannot be subtyped as surface epithelial, germ cell, sex-cord, or neuroendocrine cells. In terms of oncogenesis, approximately 80% of small cell carcinomas of ovary overexpress p53 protein, as seen in our second patient, suggesting that a *p53* mutation may be an underlying cause [14]. Similar to other malignancies, in ovarian small cell carcinoma, overexpression of p53 may not explain entirely the origin of tumorigenesis, and further investigation is needed.

## 4. Discussion

Ovarian small cell tumors are highly aggressive tumors. At least half of them have spread beyond the ovary at the time of laparotomy, but this may be an underestimation as many of the patients were not optimally staged [1]. Metastatic tumors occur mostly within the pelvis and abdomen, but hematogenous spread also occurs. The primary therapy of this tumor is unilateral salpingo-oophorectomy in stage IA, that has a reported postoperative disease-free survival rate of approximately 33% throughout 1 to 13 years. However, even at stage IA, more than 50% of patients die of their disease. Almost all patients with higher stage tumors die of disease, usually within 2 years. Effective treatment of patients with high-stage tumors or recurrent disease has not yet been achieved, although rare patients with high-stage tumors have survived over 4 years after intensive chemotherapy, radiation therapy, or both [1, 3, 4]. Potentially favorable prognostic features in stage IA cases include: age > 30 years, normal preoperative calcium level, tumor size up to 10 cm, absence of large cells, operation that included bilateral oophorectomy, and postoperative radiation therapy [1]. In the largest published series on OSCCHT, the majority of 150 patients received some form of adjuvant chemotherapy. However, only 7 patients showed a favorable response to therapy, which in their case, consisted of various regimens, including anthracyclins, etoposide, cisplatin, and alkylating agents [1]. Young et al. [1] proposed a more radical surgical therapy, including hysterectomy and bilateral salpingo-oophorectomy. However, more recent reports have indicated that a fertility sparing surgical approach, in conjugation with multiagent adjuvant chemotherapy, may not compromise the survival of these patients [5, 6, 15]. In a report by Harrison et al. [15], 17 patients received adjuvant cisplatin- and etoposide-based chemotherapy. In total, 6 of 10 patients with Stage I disease and 1 of 7 patients with Stage III disease were alive and disease-free at the time of the report. Similarly, two recent papers by Dykgraaf et al. [7] and McCormick et al. [8] reported two cases of patients who were operated and subsequently received platinum-based therapy (bleomycin, etoposide, and cisplatin in the Dykgraaf et al. paper [7] and cisplatin-etoposide in the McCormick et al. paper [8]). Dykgraaf et al. reported a substantial relapse-free survival of 60 months [7]. Another recently published paper by Nelsen et al. [9] reported a case of 25-year old female who was operated for a IIIC small cell carcinoma of the ovary. After surgery, the patient received two cycles of intensive cyclophosphamide, etoposide, and cisplatin regimen, followed by three cycles of ifosphamide, carboplatin, and etoposide and an autologous peripheral blood stem cell transplant. The patient was free of recurrence, 17 months after her initial diagnosis. 

It is worth noting that paclitaxel-based regimens, commonly used in other types of ovarian cancer, demonstrated only limited efficacy in both first line and salvage therapy [6, 15]. Harrison et al. [15] also concluded that radiotherapy had a significant additional therapeutic impact, while Baeyens et al. reported the first case that has been successfully cured by surgery plus adjuvant radiotherapy only [16].

In summary, the optimal management of OSCCHT remains unknown. Currently, a platin-based regimen that includes etoposide, alkylating agents, and anthracyclines appears to be the most promising and requires further evaluation.

## Figures and Tables

**Table 1:** Literature review of the treatment of small cell carcinoma of the ovaries.

Trial	*n*	Regimen
Young et al. [1]	150	Vincristine, vinblastine, cyclophosphamide, doxorubicin, etoposide, and cisplatin.
Chen et al. [2]	1	Paclitaxel/carboplatin followed by consolidation paclitaxel.
Tewari et al. [3]	1	Vinblastine, cisplatin, cyclophosphamide, bleomycin, doxorubicin, and etoposide.
Benrubi et al. [4]	1	Cyclophosphamide, doxorubicin, cisplatin, vincristine, and etoposide.
Rana et al. [5]	1	Vinblastine, cisplatin, cyclophosphamide, bleomycin, doxorubicin, and etoposide.
Distelmaier et al. [6]	11	Cisplatin, etoposide, ifosfamide (*n* = 4 pts) carboplatin, etoposide, vincristine, actinomycin, ifosfamide, and adriamycin (*n* = 2 pts) carboplatin/paclitaxel followed by cyclophosphamide/paclitaxel (*n* = 1 pts).
Dykgraaf et al. [7]	1	Bleomycin, etoposide, and cisplatin.
McCormick et al. [8]	1	Cisplatin and etoposide.
Nelsen et al. [9]	1	Two cycles of intensive cyclophosphamide, etoposide, and cisplatin regimen, followed by three cycles of ifosfamide, carboplatin and etoposide and an autologous peripheral blood stem cell transplant.

## References

[1] Young RH, Oliva E, Scully RE (1994). Small cell carcinoma of the ovary, hypercalcemic type: a clinicopathological analysis of 150 cases. *American Journal of Surgical Pathology*.

[2] Chen L, Dinh TA, Haque A (2005). Small cell carcinoma of the ovary with hypercalcemia and ectopic parathyroid hormone production. *Archives of Pathology and Laboratory Medicine*.

[3] Tewari K, Brewer C, Cappuccini F, Macri C, Rogers LW, Berman ML (1997). Advanced-stage small cell carcinoma of the ovary in pregnancy: long-term survival after surgical debulking and multiagent chemotherapy. *Gynecologic Oncology*.

[4] Benrubi GI, Pitel P, Lammert N (1993). Small cell carcinoma of the ovary with hypercalcemia responsive to sequencing chemotherapy. *Southern Medical Journal*.

[5] Rana S, Warren BK, Yamada SD (2004). Stage IIIC small cell carcinoma of the ovary: survival with conservative surgery and chemotherapy. *Obstetrics and gynecology*.

[6] Distelmaier F, Calaminus G, Harms D (2006). Ovarian small cell carcinoma of the hypercalcemic type in children and adolescents: a prognostically unfavorable but curable disease. *Cancer*.

[7] Dykgraaf RH, De Jong D, Van Veen M, Ewing-Graham PC, Helmerhorst TJM, Van Der Burg MEL (2009). Clinical management of ovarian small-cell carcinoma of the hypercalcemic type: a proposal for conservative surgery in an advanced stage of disease. *International Journal of Gynecological Cancer*.

[8] McCormick TC, Muffly T, Lu G, Shoup B (2009). Aggressive small cell carcinoma of the ovary, hypercalcemic type with hypercalcemia in pregnancy, treated with conservative surgery and chemotherapy. *International Journal of Gynecological Cancer*.

[9] Nelsen LL, Muirhead DM, Bell MC (2010). Ovarian small cell carcinoma, hypercalcemic type exhibiting a response to high-dose chemotherapy. *South Dakota medicine : the journal of the South Dakota State Medical Association*.

[10] Florell SR, Bruggers CS, Matlak M, Young RH, Lowichik A (1999). Ovarian small cell carcinoma of the hypercalcemic type in a 14 month old: the youngest reported case. *Medical and Pediatric Oncology*.

[11] Longy M, Toulouse C, Mage P, Chauvergne J, Trojani M (1996). Familial cluster of ovarian small cell carcinoma: a new mendelian entity?. *Journal of Medical Genetics*.

[12] Dickersin GR, Kline IW, Scully RE (1982). Small cell carcinoma of the ovary with hypercalcemia: a report of eleven cases. *Cancer*.

[13] Matias-Guiu X, Prat J, Young RH (1994). Human parathyroid hormone-related protein in ovarian small cell carcinoma: An immunohistochemical study. *Cancer*.

[14] Seidman JD (1995). Small cell carcinoma of the ovary of the hypercalcemic type: p53 protein accumulation and clinicopathologic features. *Gynecologic Oncology*.

[15] Harrison ML, Hoskins P, Du Bois A (2006). Small cell of the ovary, hypercalcemic type—analysis of combined experience and recommendation for management. A GCIG study. *Gynecologic Oncology*.

[16] Baeyens L, Amat S, Vanden Houte K, Vanhoutte P, L’Hermite M (2008). Small cell carcinoma of the ovary successfully treated with radiotherapy only after surgery: case report. *European Journal of Gynaecological Oncology*.

